# Bivariate copula regression models for semi-competing risks 

**DOI:** 10.1177/09622802231188516

**Published:** 2023-08-09

**Authors:** Yinghui Wei, Małgorzata Wojtyś, Lexy Sorrell, Peter Rowe

**Affiliations:** 1 Centre for Mathematical Sciences, School of Engineering, Computing and Mathematics, 6633 University of Plymouth, Plymouth, UK; 2 South West Transplant Centre, University Hospitals Plymouth NHS Trust, Plymouth, UK

**Keywords:** Copula model, renal transplant, semi-competing risk, survival analysis, hazard ratio

## Abstract

Time-to-event semi-competing risk endpoints may be correlated when both events occur on the same individual. These events and the association between them may also be influenced by individual characteristics. In this article, we propose copula survival models to estimate hazard ratios of covariates on the non-terminal and terminal events, along with the effects of covariates on the association between the two events. We use the Normal, Clayton, Frank and Gumbel copulas to provide a variety of association structures between the non-terminal and terminal events. We apply the proposed methods to model semi-competing risks of graft failure and death for kidney transplant patients. We find that copula survival models perform better than the Cox proportional hazards model when estimating the non-terminal event hazard ratio of covariates. We also find that the inclusion of covariates in the association parameter of the copula models improves the estimation of the hazard ratios.

## 1. Introduction

Often in medical studies, patients who are lost to follow-up, or do not experience the event of interest during the study period, leave a censored observation. However, with semi-competing risk endpoints, the non-terminal event has the possibility of also being censored by the terminal event.^1^ One example of semi-competing risks, which will be analysed later in this article, is graft failure and death following kidney transplant, where the non-terminal event is graft failure and the terminal event is death.

The Cox proportional hazards model is widely used in practice to analyse time-to-event outcomes. The Cox model was originally developed to analyse all-cause mortality,^2^ of which there is no competing risk. The hazard ratios estimated from the Cox model, assuming an independent censoring mechanism, are potentially biased when analysing a non-terminal event.^3^ Some authors use copula regression models to jointly model non-terminal and terminal events. Peng and Fine,^4^ Hsieh et al.^5^ and Chen^6^ proposed semiparametric copula models for the regression on the marginal distributions. The analysis on the terminal event can be conducted using common survival analysis methodology. For instance, Peng and Fine^4^ model the terminal event with a proportional hazards model marginally within their copula model. Hsieh et al.^5^ used a copula model, which allows for different dependence structures between covariate groups. The Bayesian normal induced copula estimation model is developed by Fu et al.^7^

Copulas are multivariate distribution functions which can model the marginal distributions separately, along with the dependence structure between them. Sorrell et al.^8^ introduced bivariate copula models to estimate the correlation between semi-competing risk endpoints, using the following four copula functions, the Normal, Clayton, Frank and Gumbel copulas. However, the hazard rates of the non-terminal and terminal events, along with the association parameter between them, may be influenced by covariates. Including covariates into the analysis of the correlation between survival endpoints can help understand how the association may be influenced by individual characteristics. Covariates may also be included in the analysis of marginal distributions, which allows the estimation of hazard ratios and subsequently the comparison of risks of survival endpoints between groups.

In this article, we develop copula survival regression models by using conditional copulas,^9^ which allow both the association parameter between the survival endpoints and the hazard rates to depend on multiple binary covariates. This is an extension from the copula survival model introduced by Sorrell et al.,^8^ where no covariates are included. We estimate the hazard ratio using copula survival regression models with binary covariates. We also estimate the effects of these binary covariates on the association between semi-competing risks. By jointly modelling the non-terminal and terminal events and including the correlation between them, we hope to improve the inference about the non-terminalevent.

We focus on the inclusion of covariates in the analysis of survival data with a semi-competing risk using conditional copulas. In the semi-competing risk setting, Chen^6^ developed a model allowing the association parameter of the copula function to vary with categorical covariates. A model allowing each covariate group to assume a separate Archimedean copula function is introduced by Hsieh et al.^5^ This allows different dependence structures describing the association between the semi-competing risk events for each covariate group. The effect of a discrete covariate on the non-terminal and terminal event and the association parameter between the semi-competing risk events is investigated by Ghosh.^10^ Peng and Fine^4^ studied the effect of a covariate on the non-terminal event and the association parameter, by proposing a model with a time-dependent copula and applying the methods to survival endpoints following AIDS diagnosis. Similarly, Zhu et al.^11^ proposed a copula regression approach to examine covariate effects on the non-terminal and terminal events using a two-stage approach. In stage 1, the covariate effects on the marginal events are estimated and in stage 2, the association parameter of the copula model is estimated. Zhu et al.^11^ prefered the two-stage approach, compared to the one-stage approach by Peng and Fine,^4^ as it allows for separate identification of misspecification of the marginal regression models and the copula model.

The rest of the article is organised as follows. The motivating data for this article are described in Section 2. We then introduce our proposed copula regression survival methods to estimate the effect of covariates on the semi-competing risk events and on the association parameter using a variety of conditional copula functions in Section 3. In Section 4, we apply our proposed methods to the motivating data, followed by a simulation study to compare the copula models in Section 5. We conclude with a discussion.

## 2. Motivating data

The motivating data are from the United Kingdom Transplant Registry (UKTR), held by the National Health Service (NHS) Blood and Transplant. The outcomes, time to graft failure and time to death since kidney transplantation, are semi-competing risks. Our study population is kidney transplant recipients, who had their single and first transplant between 1995 and 2016 in the UK, with known age, sex and donor type at the time of transplantation. We present a novel application by using our proposed copula survival regression models.

We included 40,348 single and first kidney transplants between 1995 and 2016 in the UK. For 78.0% of patients the graft failure time is censored, for 80.0% of patients the death time is censored and for 64.3% of patients both events are censored.

Table 1 shows the baseline characteristics of interest. The covariates to be included in the application later in this article are donor type (deceased or living), recipient’s sex (male or female), and recipient’s age ( >50 years or ≤50 years). Donor type indicates whether a recipient had received a deceased donor or living donor kidney for their transplantation. These covariates were included as they are important factors influencing transplant outcomes according to the literature as follows. As reported by Terasaki et al.^12^ and Port et al.,^13^ living kidney donor recipients have improved survival compared to deceased donor recipients. Moreover, recipient’s age has been shown to be a factor affecting survival following transplant.^14–16^ In Fabrizii et al.,^15^ the five-year survival was found to be affected by recipient age group, however, these differences were not evident after controlling for confounding.

**Table 1.** Baseline characteristics for single and first kidney transplant recipients from the UK Transplant Registry data set between 1995 and 2016. The number of recipients ( n ) with the particular characteristic is given alongside the percentage ( % ) in brackets.

Variable		n(%)
Donor type	Deceased	27,971 ( 69.3 )
	Living	12,377 ( 30.7 )
Recipient’s sex	Male	24,945 ( 61.8 )
	Female	15,403 ( 38.2 )
Recipient’s age (years)	≤50	23,208 ( 57.5 )
	>50	17,140 ( 42.5 )

## 3. Methods

In this section, we describe methods to include covariates in copula functions to examine the effects that they have on the semi-competing risk events. We let the association parameter of the copula function, along with the hazard rates from the marginal survival distributions be conditional on covariates. We use conditional copulas, first introduced by Patton^9^ who extended Sklar’s theorem^17^ to account for the conditioning of the random variables on covariates.

Let $T_{1}$ denote the time to the non-terminal event and $T_{2}$ denote the time to the terminal event with censoring time, C . The time to the first event is denoted by $X=\min(T_{1},T_{2},C)$ with event indicator $d_{1}=1$ if $T_{1}\leq \min(T_{2},C)$ and $d_{1}=0$ otherwise. The time to the second event is denoted by $Y=\min(T_{2},C)$ with event indicator $d_{2}=1$ if $T_{2}\leq C$ and $d_{2}=0$ , otherwise.

We let the marginal hazard rates and the association parameter depend on covariates, $W=(W_{1},\ldots ,W_{p})$ . The marginal survival functions are given by $S_{T_{1}|W}(t_{1}|\text{w})=P(T_{1}>t_{1}|\text{w})$ and $S_{T_{2}|W}(t_{2}|\text{w})=P(T_{2}>t_{2}|\text{w})$ for the non-terminal and terminal events, respectively. The marginal probability density functions (PDFs) are denoted by $f_{T_{1}|W}(t_{1}|\text{w})$ and $f_{T_{2}|W}(t_{2}|\text{w})$ for the non-terminal and terminal events, respectively.

We estimate the association between the survival probabilities of individuals, using univariate survival functions in the copula function. This changes the interpretation of association between the endpoints,^18^ compared to the use of the cumulative distribution function (CDF) by Patton.^9^ We represent the joint survival function using the copula function, $C_{\theta }$ , with association parameter θ ,

(1) $$S_{B}(t_{1},t_{2}|\text{w})=P(T_{1}>t_{1},T_{2}>t_{2}|\text{w})=C_{\theta }(S_{T_{1}|W}(t_{1}|\text{w}),S_{T_{2}|W}(t_{2}|\text{w})|\text{w})$$

where the subscript B is used to indicate the bivariate nature of the function.

Then, the copula density function is as follows

(2) $$c_{\theta }(S_{T_{1}|W}(t_{1}|\text{w}),S_{T_{2}|W}(t_{2}|\text{w}))=\frac{\partial^{2}C_{\theta }(S_{T_{1}|W}(t_{1}|\text{w}),S_{T_{2}|W}(t_{2}|\text{w})|\text{w})}{\partial S_{T_{1}|W}(t_{1}|\text{w})\partial S_{T_{2}|W}(t_{2}|\text{w})}$$

The joint PDF can be expressed using the copula density function and the marginal PDFs,

(3) $$f_{B}(t_{1},t_{2}|\text{w})=c_{\theta }(S_{T_{1}|W}(t_{1}|\text{w}),S_{T_{2}|W}(t_{2}|\text{w})|\text{w})f_{T_{1}|W}(t_{1}|\text{w})f_{T_{2}|W}(t_{2}|\text{w})$$

We consider four different types of copula, the Normal, Clayton, Frank and Gumbel copulas, for which the definitions are given by Sorrell et al.^8^ These copula functions cover a range of dependence structures and offer different shapes of the joint survival function in equation (1). For each of these copulas, the association parameter θ is univariate. For the marginal distributions for both events, we consider the Exponential, Weibull and Gompertz distributions, which are commonly used parametric survival models. We use them to illustrate the methods and applications, however, these can be easily extended to other parametric survival distributions.

### 3.1. Likelihood

Let Θ be a vector of the parameters of the marginal distributions and the association parameter θ that are conditional on covariates. Then, the likelihood function is given as follows:

(4) $$\begin{array}{rl} L(\Theta )= & \prod_{i=1}^{n}{\left(c_{\theta }(S_{T_{1}|W}(t_{i,1}|{\text{w}}_{i}),S_{T_{2}|W}(t_{i,2}|{\text{w}}_{i})|{\text{w}}_{i})f_{T_{1}|W}(t_{i,1}|{\text{w}}_{i})f_{T_{2}|W}(t_{i,2}|{\text{w}}_{i})\right)}^{d_{i,1}d_{i,2}}\\ & \;\times {\left(\frac{\partial C_{\theta }(S_{T_{1}|W}(t_{i,1}|{\text{w}}_{i}),S_{T_{2}|W}(t_{i,2}|{\text{w}}_{i})|{\text{w}}_{i})}{\partial S_{T_{1}|W}(t_{i,1}|{\text{w}}_{i})}f_{T_{1}|W}(t_{i,1}|{\text{w}}_{i})\right)}^{d_{i,1}(1-d_{i,2})}\\ & \;\times {\left(\frac{\partial C_{\theta }(S_{T_{1}|W}(t_{i,1}|{\text{w}}_{i}),S_{T_{2}|W}(t_{i,2}|{\text{w}}_{i})|{\text{w}}_{i})}{\partial S_{T_{2}|W}(t_{i,2}|{\text{w}}_{i})}f_{T_{2}|W}(t_{i,2}|{\text{w}}_{i})\right)}^{(1-d_{i,1})d_{i,2}}\\ & \;\times {\left(C_{\theta }(S_{T_{1}|W}(t_{i,1}|{\text{w}}_{i}),S_{T_{2}|W}(t_{i,2}|{\text{w}}_{i})|{\text{w}}_{i})\right)}^{\left(1-d_{i,1})(1-d_{i,2}\right)} \end{array}$$

where n is the total number of individuals.

Below, we give the formulae for the likelihood when the four types of copula, the Normal, Clayton, Frank and Gumbel, are used. Table 2 summarises the different link functions for each copula that are used to ensure the association parameters are within the permissible ranges within the respective copula functions.

**Table 2.** Link functions for the association parameter θ .

Copula $C_{\theta }$	Link function for θ	Range of θ
Normal	$\theta =\frac{\exp\;(2(b_{0}+b_{1}W_{1}+\cdots +b_{p}W_{p}))-1}{\exp\;(2(b_{0}+b_{1}W_{1}+\cdots +b_{p}W_{p}))+1}$	θ∈[−1,1]
Clayton	$\theta =\exp\;(b_{0}+b_{1}W_{1}+\cdots +b_{p}W_{p})$	θ∈(0,∞)
Frank	$\theta =b_{0}+b_{1}W_{1}+\cdots +b_{p}W_{p}$	θ∈(−∞,∞)
Gumbel	$\theta =\exp\;(b_{0}+b_{1}W_{1}+\cdots +b_{p}W_{p})+1$	θ∈[1,∞)

For the Normal copula, the association parameter θ=ρ , where ρ is the Pearson correlation coefficient, and the likelihood function of equation (4) is given by

(5) LN(Θ)=∏i=1n(11−ρ2exp(12(1−ρ2)×2ρΦ−1(ST1|W(ti,1|wi))Φ−1(ST2|W(ti,2|wi))−ρ2(Φ−1(ST1|W(ti,1|wi))2+Φ−1(ST2|W(ti,2|wi))2)12(1−ρ2))fT1|W(ti,1|wi)fT2|W(ti,2|wi)11−ρ2)di,1di,2×(Φ(Φ−1(ST2|W(ti,2|wi))−ρΦ−1(ST1|W(ti,1|wi))1−ρ2)fT1|W(ti,1|wi))di,1(1−di,2)×(Φ(Φ−1(ST1|W(ti,1|wi))−ρΦ−1(ST2|W(ti,2|wi))1−ρ2)fT2|W(ti,2|wi))(1−di,1)di,2×(Cρ(ST1|W(ti,1|wi),ST2|W(ti,2|wi)|wi))(1−di,1)(1−di,2)

where Φ is the CDF of the standard normal distribution.

For the Clayton copula, the likelihood function is given by

(6) LC(Θ)=∏i=1n((1+θ)(Cθ(ST1|W(ti,1|wi),ST2|W(ti,2|wi)|wi))1+2θ(ST1|W(ti,1|wi)ST2|W(ti,2|wi))1+θ×fT1|W(ti,1|wi)fT2|W(ti,2|wi)(Cθ(ST1|W(ti,1|wi),ST2|W(ti,2|wi)|wi))1+2θ(ST1|W(ti,1|wi)ST2|W(ti,2|wi))1+θ)di,1di,2×((Cθ(ST1|W(ti,1|wi),ST2|W(ti,2|wi)|wi)ST1|W(ti,1|wi))1+θfT1|W(ti,1|wi))di,1(1−di,2)×((Cθ(ST1|W(ti,1|wi),ST2|W(ti,2|wi)|wi)ST2|W(ti,2|wi))1+θfT2|W(ti,2|wi))(1−di,1)di,2×(Cθ(ST1|W(ti,1|wi),ST2|W(ti,2|wi)|wi))(1−di,1)(1−di,2)

For the Frank copula, the likelihood function is given by

(7) LF(Θ)=∏i=1n(θeθCθ(ST1|W(ti,1|wi),ST2|W(ti,2|wi)|wi)(eθCθ(ST1|W(ti,1|wi),ST2|W(ti,2|wi)|wi)−1)(eθST1|W(ti,1|wi)−1)(eθST2|W(ti,2|wi)−1)×fT1|W(ti,1|wi)fT2|W(ti,2|wi)(Cθ(ST1|W(ti,1|wi),ST2|W(ti,2|wi)|wi))1+2θ(ST1|W(ti,1|wi)ST2|W(ti,2|wi))1+θ)di,1di,2×(1−eθCθ(ST1|W(ti,1|wi),ST2|W(ti,2|wi)|wi)1−eθST1|W(ti,1|wi)fT1|W(ti,1|wi))di,1(1−di,2)×(1−eθCθ(ST2|W(ti,2|wi),ST2|W(ti,2|wi)|wi)1−eθST2|W(ti,2|wi)fT2|W(ti,2|wi))(1−di,1)di,2×(Cθ(ST1|W(ti,1|wi),ST2|W(ti,2|wi)|wi))(1−di,1)(1−di,2)

Finally, for the Gumbel copula the likelihood function is given by

(8) LG(Θ)=∏i=1n(1ST1|W(ti,1|wi)ST2|W(ti,2|wi)(−log(Cθ(ST1|W(ti,1|wi),ST2|W(ti,2|wi)|wi)))2θ−1×Cθ(ST1|W(ti,1|wi),ST2|W(ti,2|wi)|wi)(−log(ST1|W(ti,1|wi)))θ−1(−log(ST2|W(ti,2|wi)))θ−1×(θ−1−log(Cθ(ST1|W(ti,1|wi),ST2|W(ti,2|wi)|wi)))fT1|W(ti,1|wi)fT2|W(ti,2|wi)(Cθ(ST1|W(ti,1|wi),ST2|W(ti,2|wi)|wi))1+2θ(ST1|W(ti,1|wi)ST2|W(ti,2|wi))1+θ)di,1di,2×(Cθ(ST1|W(ti,1|wi),ST2|W(ti,2|wi)|wi)(−log(ST1|W(ti,1|wi)))θ−1ST1|W(ti,1|wi)(−log(Cθ(ST1|W(ti,1|wi),ST2|W(ti,2|wi)|wi)))θ−1fT1|W(ti,1|wi))di,1(1−di,2)×(Cθ(ST2|W(ti,2|wi),ST2|W(ti,2|wi)|wi)(−log(ST2|W(ti,2|wi)))θ−1ST2|W(ti,2|wi)(−log(Cθ(ST2|W(ti,2|wi),ST2|W(ti,2|wi)|wi)))θ−1fT2|W(ti,2|wi))(1−di,1)di,2×(Cθ(ST1|W(ti,1|wi),ST2|W(ti,2|wi)|wi))(1−di,1)(1−di,2)

For the Normal, Clayton and Gumbel copulas, the variance of the estimator of the association parameter θ can be approximated using the Delta method. We present the derivations for the case of one covariate as well as for several covariates in the Supplemental Materials, in Section 1.2 for the Normal, Section 1.3 for the Clayton and Section 1.4 for the Gumbel copulas, respectively.

### 3.2. Event times models

We consider the Exponential, Weibull and Gompertz distributions for the marginal distributions of event times. For each model, we specify the link function for marginal parameters. An Exponential model assumes a constant hazard rate, while the Weibull and Gompertz models allow for increasing, constant or decreasing hazard rates. Our proposed methods can be easily extended to other parametric survival distributions such as a Log-normal or Log-logistic distributions.

**Exponential event times** Assume that the marginal distributions for both events follow the Exponential distribution with hazard rates $\lambda_{1}$ and $\lambda_{2}$ for the non-terminal and terminal events, respectively. Then, the survival functions are given by $S_{T_{1}|\text{W}}(t_{1})=\exp\;(-\lambda_{1}t_{1})$ and $S_{T_{2}|\text{W}}(t_{2})=\exp\;(-\lambda_{2}t_{2})$ for the non-terminal and terminal events, respectively. We incorporate covariates $W_{1},\ldots ,W_{p}$ into the hazard rates of both events:

(9) $$\begin{array}{rl} \lambda_{1} & =\exp\;(a_{0}+a_{1}W_{1}+\cdots +a_{p}W_{p}) \end{array}$$

(10) $$\begin{array}{rl} \lambda_{2} & =\exp\;(c_{0}+c_{1}W_{1}+\cdots +c_{p}W_{p}) \end{array}$$

where $a_{0},\ldots ,a_{p}$ are regression coefficients for the non-terminal event and $c_{0},\ldots ,c_{p}$ are regression coefficients for the terminal event. The hazard ratios of a binary covariate $W_{k}$ , where k∈{1,…,p} , are given by

(11) $$\begin{array}{rl} {\text{HR}}_{NT} & =\frac{\exp\;(a_{0}+a_{k})}{\exp\;(a_{0})}=\exp\;(a_{k}) \end{array}$$

(12) $$\begin{array}{rl} {\text{HR}}_{T} & =\frac{\exp\;(c_{0}+c_{k})}{\exp\;(c_{0})}=\exp\;(c_{k}) \end{array}$$

for the non-terminal (NT) and terminal (T) events, respectively.

**Weibull event times**

Assume that the marginal distributions for both events follow the Weibull distributions with the PDFs

$$f_{T_{j}|\text{W}}(t_{j})=\beta_{j}\alpha_{j}t_{j}^{\alpha_{j}-1}\exp\;(-\beta_{j}t_{j}^{\alpha_{j}})$$

for j=1,2 , where $\alpha_{j}$ is the shape parameter and $\beta_{j}$ is the scale parameter and $t_{j}$ represents the event time for the non-terminal and terminal events, respectively.

The survival function and hazard function are given as follows

$$\begin{array}{rl} S_{T_{j}|\text{W}}(t_{j}) & =\exp\;(-\beta_{j}t_{j}^{\alpha_{j}})\\ h_{T_{j}|\text{W}}(t_{j}) & =\beta_{j}\alpha_{j}t_{j}^{\alpha_{j}-1} \end{array}$$

for j=1,2 .

We consider the case where the shape parameters $\alpha_{1}$ and $\alpha_{2}$ are constant and the scale parameters $\beta_{1}$ and $\beta_{2}$ depend on the covariates $\text{W}=(W_{1},\ldots ,W_{p})$ using the following functions:

$$\begin{array}{rl} \beta_{1} & =\exp\;(a_{0}+a_{1}W_{1}+\cdots +a_{p}W_{p})\\ \beta_{2} & =\exp\;(c_{0}+c_{1}W_{1}+\cdots +c_{p}W_{p}) \end{array}$$

where $a_{0},\ldots ,a_{p}$ are regression coefficients for the non-terminal event and $c_{0},\ldots ,c_{p}$ are regression coefficients for the terminal event.

Therefore, the hazard ratios for a binary covariate $W_{k}$ , where k∈{1,…,p} , are given by

$$\begin{array}{rl} {\text{HR}}_{NT} & =\frac{\exp\;(a_{0}+a_{k})\alpha_{1}t^{\alpha_{1}-1}}{\exp\;(a_{0})\alpha_{1}t^{\alpha_{1}-1}}=\exp\;(a_{k})\\ {\text{HR}}_{T} & =\frac{\exp\;(c_{0}+c_{k})\alpha_{2}t^{\alpha_{2}-1}}{\exp\;(c_{0})\alpha_{2}t^{\alpha_{2}-1}}=\exp\;(c_{k}) \end{array}$$

respectively. These are in the same form as for the Exponential event times described in equations (11) and (12).

**Gompertz event times**

Assume that the marginal distributions for both events follow the Gompertz distributions with the PDFs

$$f_{T_{j}|\text{W}}(t_{j})=\lambda_{j}\exp\;\left(\gamma_{j}t_{j}-\frac{\lambda_{j}}{\gamma_{j}}(\exp\;(\gamma_{j}t_{j})-1)\right)$$

for j=1,2 , where $\gamma_{j}$ is the shape parameter and $\lambda_{j}$ is the rate parameter and $t_{j}$ represents the event time for the non-terminal and terminal events, respectively.

The survival function and hazard function are given as follows

$$\begin{array}{rl} S_{T_{j}|\text{W}}(t_{j}) & =\exp\;\left(-\frac{\lambda_{j}}{\gamma_{j}}(\exp\;(\gamma_{j}t_{j})-1)\right)\\ h_{T_{j}|\text{W}}(t_{j}) & =\lambda_{j}\exp\;(\gamma_{j}t_{j}) \end{array}$$

for j=1,2 .

We consider the case where the shape parameters $\gamma_{1}$ and $\gamma_{2}$ are constant and the rate parameters $\lambda_{1}$ and $\lambda_{2}$ depend on the covariates $W_{1},\ldots ,W_{p}$ using the following functions:

$$\begin{array}{rl} \lambda_{1} & =\exp\;(a_{0}+a_{1}W_{1}+\cdots +a_{p}W_{p})\\ \lambda_{2} & =\exp\;(c_{0}+c_{1}W_{1}+\cdots +c_{p}W_{p}) \end{array}$$

where $a_{0},\ldots ,a_{p}$ are regression coefficients for the non-terminal event and $c_{0},\ldots ,c_{p}$ are regression coefficients for the terminal event.

Therefore, the hazard ratios for a binary covariate $W_{k}$ , where k∈{1,…,p} , are

$$\begin{array}{rl} {\text{HR}}_{NT} & =\frac{\exp\;(a_{0}+a_{k})\exp\;(\gamma_{1}t)}{\exp\;(a_{0})\exp\;(\gamma_{1}t)}=\exp\;(a_{k})\\ {\text{HR}}_{T} & =\frac{\exp\;(c_{0}+c_{k})\exp\;(\gamma_{2}t)}{\exp\;(c_{0})\exp\;(\gamma_{2}t)}=\exp\;(c_{k}) \end{array}$$

respectively. These are in the same form as for the Exponential and Weibull event times.

For all three marginal models, the variances of the hazard ratios can be approximated by

(13) $$\begin{array}{rl} \text{Var}({\text{HR}}_{NT}) & =\exp\;(2{\hat{a}}_{k})\text{Var}({\hat{a}}_{k}) \end{array}$$

(14) $$\begin{array}{rl} \text{Var}({\text{HR}}_{T}) & =\exp\;(2{\hat{c}}_{k})\text{Var}({\hat{c}}_{k}) \end{array}$$

using the Delta method described in Section 1.1 in the Supplemental Materials.

### 3.3. Estimation

The regression coefficients for the marginal distributions and the association parameters are estimated by maximising the log-likelihood, that is, the logarithm of (4). This is achieved by using the function optim in package R^19^ in the practical application presented in this article. The 95% confidence intervals (CIs) are constructed using the Fisher information matrix.

## 4. Application

We apply the methods described in Section 3 to the UKTR data set introduced in Section 2. The data set contains time to graft failure and time to death of kidney transplant recipients. To illustrate the use of the methods, we include the following binary covariates, recipient age group ( >50 years and ≤50 years), recipient sex (female and male) and donor type (living and deceased donors), in the analysis of both survival endpoints. We allow both the hazard rates and the association between the survival endpoints to vary with these covariates. We estimate the hazard ratios of the non-terminal and terminal events along with the regression coefficients of the covariates for the association parameter between graft failure and death.

We use Exponential, Weibull and Gompertz distributions to model the survival time. We apply four different copula functions to describe the association between the survival endpoints and we select the best fitting models using the Akaike information criterion (AIC).^20^ The estimated hazard ratios for each covariate and the regression coefficients of the covariates for the association parameter are provided in Tables 3 to 5 for the Exponential, Gompertz and Weibull survival distributions, respectively. The results for the Cox model are presented in Table 3. Moreover, in Tables 3 to 5 the computational time for each model is reported for a computer with processor $11^{\mathrm{th}}$ Gen Intel (R) Core(TM) i7-1165G7 CPU @ 2.80 GHz.

**Table 3.** Cox model and Exponential survival copula models: Results from analysing the UK Transplant Registry data set. The binary covariates are defined as follows, sex: female versus male (reference), donor type: living versus deceased (reference), and age group: >50 years versus ≤50 years (reference).

	Hazard ratio (95% CI)		Regression coefficients on		Time
Model/covariate	Graft failure (GF)	Death (D)	association parameter	AIC	(minutes)
Cox model				174,873.6 (GF)	0.004
				153,814.9 (D)	0.005
Age group: >50	0.911 (0.872, 0.952)	3.926 (3.745, 4.116)	NA		
Sex: Female	1.029 (0.986, 1.073)	0.881 (0.841, 0.922)	NA		
Donor type: Living	0.615 (0.584, 0.648)	0.539 (0.506, 0.574)	NA		
Normal copula Exponential survival model				147545.5	123.60
Age group: >50	1.121 (1.074, 1.169)	3.748 (3.576, 3.920)	0.285 (0.248, 0.321)		
Sex: Female	1.015 (0.973, 1.058)	0.900 (0.860, 0.939)	0.021 (−0.015, 0.058)		
Donor type: Living	0.600 (0.569, 0.630)	0.522 (0.490, 0.553)	0.027 (−0.025, 0.078)		
Clayton copula Exponential survival model				146956.7	1.63
Age group: >50	1.383 (1.329, 1.437)	3.863 (3.691, 4.036)	0.093 (0.983, 1.203)	
Sex: Female	1.006 (0.967, 1.045)	0.934 (0.898, 0.969)	0.139 (0.039, 0.239)	
Donor type: Living	0.587 (0.559, 0.616)	0.540 (0.512, 0.568)	0.529 (0.384, 0.674)	
Frank copula Exponential survival model				146660.5	2.19
Age group: >50	1.361 (1.309, 1.414)	3.861 (3.689, 4.032)	5.068 (4.600, 5.537)	
Sex: Female	1.000 (0.961, 1.039)	0.930 (0.893, 0.966)	0.352 (−0.059, 0.762)	
Donor type: Living	0.586 (0.557, 0.615)	0.535 (0.506, 0.564)	0.861 (0.211, 1.511)		
Gumbel copula Exponential survival model				147827.8	4.29
Age group: >50	1.134 (1.086, 1.182)	3.667 (3.498, 3.837)	1.355 (1.204, 1.507)		
Sex: Female	1.015 (0.973, 1.057)	0.899 (0.859, 0.939)	0.056 (−0.079, 0.191)		
Donor type: Living	0.603 (0.573, 0.634)	0.530 (0.498, 0.562)	−0.039 (−0.231, 0.153)		

**Table 4.** Gompertz survival copula models: Results from analysing the UK Transplant Registry data set. The binary covariates are defined as follows, sex: female versus male (reference), donor type: living versus deceased (reference), and age group: >50 years versus ≤50 years (reference).

	Hazard ratio (95% CI)				
			Regression coefficients on		Time
Model/covariate	Graft failure	Death	association prameter	AIC	(minutes)
Normal copula Gompertz survival model				146872.6	250.8
Age group: >50	1.080 (1.033, 1.126)	4.163 (3.969, 4.357)	0.282 (0.246, 0.318)		
Sex: Female	1.018 (0.976, 1.061)	0.893 (0.854, 0.932)	0.024 (−0.012, 0.060)		
Donor type: Living	0.594 (0.563, 0.624)	0.553 (0.520, 0.586)	0.020 (−0.03, 0.071)		
Clayton copula Gompertz survival model				146708.3	5.28
Age group: >50	1.296 (1.242, 1.349)	3.935 (3.756, 4.114)	0.947 (0.835, 1.059)		
Sex: Female	0.999 (0.959, 1.039)	0.909 (0.873, 0.945)	0.161 (0.059, 0.263)		
Donor type: Living	0.592 (0.562, 0.622)	0.555 (0.524, 0.586)	0.287 (0.118, 0.456)		
Frank copula Gompertz survival model				146409.4	4.81
Age group: >50	1.232 (1.182, 1.283)	3.949 (3.771, 4.127)	2.070 (1.655, 2.486)		
Sex: Female	1.003 (0.963, 1.042)	0.919 (0.881, 0.957)	0.639 (0.233, 1.045)		
Donor type: Living	0.544 (0.516, 0.572)	0.498 (0.469, 0.528)	0.423 (−0.240, 1.087)		
Gumbel copula Gompertz survival model				147036.8	6.67
Age group: >50	1.065 (1.020, 1.111)	4.110 (3.918, 4.303)	1.401 (1.251, 1.505)		
Sex: Female	1.012 (0.970, 1.054)	0.891 (0.852, 0.931)	−0.017 (−0.152, 0.118)		
Donor type: Living	0.596 (0.566, 0.627)	0.557 (0.524, 0.591)	−0.096 (−0.290, 0.097)		

**Table 5.** Weibull survival copula models: Results from analysing the UK Transplant Registry data set. The binary covariates are defined as follows, sex: female versus male (reference), donor type: living versus deceased (reference), and age group: >50 years versus ≤50 years (reference).

	Hazard ratio (95% CI)		Regression coefficients		Time
Model/covariate	Graft failure	Death	on association parameter	AIC	(minutes)
Normal copula Weibull survival model				145038.4	201.00
Age group: >50	1.005 (0.962, 1.048)	3.692 (3.523, 3.860)	0.283 (0.240, 0.326)		
Sex: Female	1.022 (0.979, 1.064)	0.904 (0.865, 0.943)	0.028 (−0.014, 0.071)		
Donor type: Living	0.573 (0.544, 0.602)	0.520 (0.489, 0.551)	0.034 (−0.027, 0.094)		
Clayton copula Weibull survival model				145137.8	8.03
Age group: >50	1.212 (1.160, 1.264)	3.699 (3.532, 3.867)	0.907 (0.782, 1.032)		
Sex: Female	1.024 (0.983, 1.066)	0.924 (0.887, 0.96)	0.162 (0.054, 0.271)		
Donor type: Living	0.562 (0.533, 0.590)	0.527 (0.498, 0.556)	0.479 (0.314, 0.643)		
Frank copula Weibull survival model				144704.7	6.16
Age group: >50	1.202 (1.152, 1.251)	3.734 (3.565, 3.903)	3.954 (3.488, 4.402)		
Sex: Female	1.005 (0.965, 1.045)	0.920 (0.882, 0.957)	0.171 (−0.251, 0.592)		
Donor type: Living	0.560 (0.532, 0.588)	0.519 (0.490, 0.549)	0.493 (−0.174, 1.160)		
Gumbel copula Weibull survival model				145276.1	6.27
Age group: >50	0.992 (0.949, 1.034)	3.600 (3.436, 3.765)	1.073 (0.935, 1.211)		
Sex: Female	1.020 (0.978, 1.062)	0.906 (0.867, 0.945)	0.044 (−0.08, 0.169)		
Donor type: Living	0.581 (0.552, 0.611)	0.520 (0.489, 0.551)	−0.043 (−0.218, 0.132)		

### 4.1. Hazard ratios

#### 4.1.1. Graft failure following transplant

Across all considered models, sex is not found to be associated with the risk for graft failure. Compared to a deceased donor transplant, living donor transplant is associated with lower risk for graft failure. Older age ( >50 years) is found to be associated with increased risk across all considered copula models except for two cases: Normal and Gumbel copulas with Weibull survival model, where there is no association found. This is in contrast with the Cox model where the older age group is found to be associated with lower risk of graft failure (HR: 0.911, 95% CI: 0.872–0.952, Table 3). This may be in part due to the censoring of graft failure by death in the Cox model, the older individuals who die before experiencing graft failure may be seen as less likely to experience graft failure.

In the fitted Cox model for graft failure, there was evidence for non-proportional hazards for all three covariates. Using Grambsch and Therneau’s approach^21^ to diagnose non-proportionality, we obtained *P*-values of 0.091, 0.017 and <0.001 for sex, age and donor type, respectively.

#### 4.1.2. Death following transplant

For all considered models, including the Cox model and all copula models, all three covariates: sex, age and donor type, are found to be associated with death after transplant. In particular, female sex, living donor transplant and younger age ( ≤50 years) are associated with lower risk of death. In contrast, male sex, deceased donor transplant and older age ( >50 years) are associated with higher risk of death. These findings are consistent across all models and the estimated hazard ratios are fairly similar.

In the fitted Cox model for death, there was evidence for non-proportional hazards for donor type ( P<0.001 ), whilst there was insufficient evidence for non-proportional hazards for sex ( P=0.936 ) and age ( P=0.882 ).

### 4.2. Association between graft failure and death

The results from all the copula survival models showed that the association between graft failure and death is stronger for individuals in the older age group compared to the younger age group.

In most of the fitted copula models, we observed no difference between female and male recipients for the association between graft failure and death. The exceptions are the Clayton copula models and Frank copula Gompertz model which show stronger association between these two end points for female recipients. Similarly, in most of the fitted copula models, we observed no difference between living and deceased donors for the association between graft failure and death. The exceptions are the Clayton copula models and Frank copula Exponential model which showed stronger association between these two end points for living donor recipients.

### 4.3. Results for the preferred model

In our real data analyses, we reported the AIC value for each model. However, we did not compare Cox model with the copula models using AIC. This is because the Cox model was fitted to graft failure and death, separately, and the Cox model has a partial likelihood instead of a full likelihood. In contrast, the copula survival model analysed graft failure and death jointly, and has a full likelihood. Hence, the AIC values for the Cox model and the copula-based parametric survival models are not comparable. However, we used the AIC to compare between the copula survival models. For each survival distribution, the Frank copula model is preferred according to the AIC (Tables 3 to 5). Moreover, for each copula model, the Weibull survival distribution provides the lowest AIC.

In the Frank copula Weibull survival model, the association between graft failure and death is affected by age, with individuals in the older age group having a stronger association compared to those in the younger age group. Living donor recipient group is at lower risk for graft failure and death, with respective hazard ratios 0.560 (95% CI: 0.532–0.588) and 0.519 (95% CI: 0.490–0.549). Female sex is associated with lower risk for death compared to men, with hazard ratio 0.920 (95% CI: 0.882–0.957). The hazard ratio of graft failure for the older age group is 1.202 (95% CI: 1.152–1.251) and the hazard ratio of death is 3.734 (95% CI: 3.565–3.903), indicating the older age group is at higher risk of graft failure and death.

**Figure 1.**
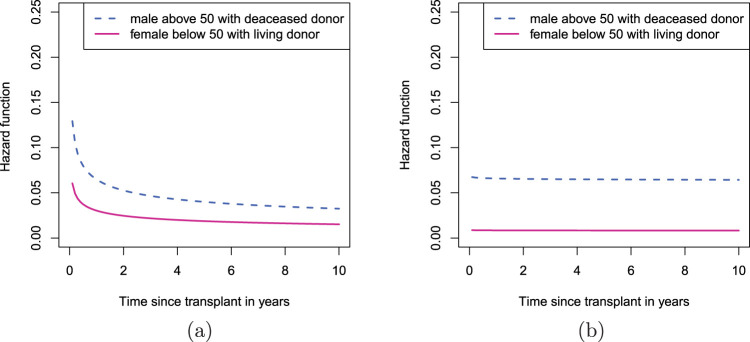
Estimated hazard functions for two subgroups of patients: male recipients above 50 years with deceased donor and female recipients aged below 50 years with living donor, using Frank copula with Weibull survival times: (a) graft failure; (b) death.

The Frank copula Weibull survival model suggests that in general male patients above 50 years who received a transplant from a deceased donor are at highest risk for death following kidney transplant. At the other extreme, younger female patients who received a transplant from a living donor are at the lowest risk for death. The hazard functions estimated from the preferred model are presented in Figure 1 for these two subgroups, respectively. For both subgroups, the hazard for graft failure is greatest immediately following the transplant and gradually decreases within 4 years where it is stabilised (Figure 1(a)). The hazard for death is stable over time since transplant (Figure 1(b)). For male recipients aged >50 years with a deceased donor, for the first 2 years following kidney transplant, the hazard for graft failure is greater than for death and after that the order is reversed.

## 5. Simulation study

We conduct two simulation studies. In simulation study 1, we aim to assess the performance of the proposed bivariate copula regression models for semi-competing risk data. We simulate data to mimic the real example of renal transplant data. We compare the performance of three models: the Cox proportional hazards model; the bivariate copula regression models with predictors for the hazard rates (copula model 1); and the bivariate copula regression models with predictors for both hazard rates and the association parameters (copula model 2).

In simulation study 2, we aim to assess the effect of misspecification of the survival distributions on the estimation of the model parameters. We use the AIC value to select the best fitting model for the simulated data and calculate the percentage that the underlying survival model is correctly selected.

### 5.1. Design

We simulate data with known marginal hazard rates for the non-terminal and terminal events, and the correlation between the two event times. We assess the accuracy of the estimates for the hazard ratios of the non-terminal and terminal events, ${\text{HR}}_{NT}$ and ${\text{HR}}_{T}$ , respectively. The number of replications in both simulation studies was 1000. The simulation algorithm is provided below.

1. We generate the binary covariates, $W_{k}$ , where k=1,…,p for p covariates, in proportions that represent the renal transplant data.
2. We generate Pearson’s correlation coefficient for the Normal copula, or association parameters for the Clayton, Frank and Gumbel copulas, respectively, as described in Table 2, with chosen $b_{k}$ .
3. Hazard rates for the non-terminal event, $\lambda_{1}$ , and terminal event $\lambda_{2}$ , with chosen $a_{k}$ and $c_{k}$ , are generated by using equations (9) and (10), respectively.
4. We use the conditional distribution method^22^ to simulate time to the non-terminal and terminal events from a specified copula.
  -
(U,V) have the joint distribution function of the chosen copula, where U represents the uniformly transformed time to the non-terminal event, and V represents the uniformly transformed time to the terminal event.
  - Generate U from Uniform(0,1) .
  - Generate V from $C^{-1}(v|u)$ , which is the inverse of the conditional copula distribution function of V given U .
5. We obtain $T_{1}$ and $T_{2}$ from the respective inverse marginal survival functions,
$$\begin{array}{rl} T_{1} & =S_{T_{1}}^{-1}(U),\\ T_{2} & =S_{T_{2}}^{-1}(V). \end{array}$$
  Here $T_{1}=\frac{-\log\;(U)}{\lambda_{1}}$ and $T_{2}=\frac{-\log\;(V)}{\lambda_{2}}$ , when the survival distribution is Exponential for both events.
6. We simulate censoring time, C , independently from a uniform distribution.
7. Set the time to the non-terminal event, $X=\min(T_{1},T_{2},C)$ , with event indicator $d_{1}=1$ if $T_{1}\leq \min(T_{2},C)$ and $d_{1}=0$ , otherwise.
8. Set the time to the terminal event, $Y=\min(T_{2},C)$ , with event indicator $d_{2}=1$ if $T_{2}\leq C$ and $d_{2}=0$ , otherwise.

In simulation study 1, the simulated data are analysed using Cox model, and the models with underlying copula function. For example, when analysing the simulated data generated using the Clayton copula, we use the Clayton copula model. Sorrell et al.^8^ conducted an extensive simulation study to evaluate the effect of misspecification of copula functions and we will not duplicate that simulation in this article.

### 5.2. Choice of parameters

We generate data sets with 3000 individuals with hazard rates and association parameters mimicking the real data analysis from the UKTR data set. The binary covariates are generated from a Bernoulli distribution with parameters mimicking the real data. Specifically, the age group >50 years with probability 0.40, females with probability 0.38 and living donor recipients with probability 0.30.

The regression coefficients are set to be equal to those in Supplemental Materials Tables S1 and S2 for simulation studies 1 and 2, respectively. The censoring time, C , is generated independently from Uniform(0,25) distribution, representing a maximum follow-up time of 25 years.

### 5.3. Performance measures

We maximise the log-likelihood described in Section 3 using the optim function in R to estimate the regression coefficients for the marginal and association parameters. The performance of the maximum likelihood estimates is evaluated using the bias, the mean squared error (MSE) and the coverage probability.

### 5.4. Results of simulation study 1: Performance of the proposed bivariate copula regression models

For the non-terminal event, the hazard ratio of age group estimated from the Cox model has the following coverage probabilities, 11.7% , 0.0% , 0.0% and 18.9% , if data were simulated from Normal, Clayton, Frank and Gumbel copulas, respectively (Table 6). These coverage probabilities are far below the nominal level. In copula regression model with covariates included for the hazard rate but not for the association parameter (copula model 1), the equivalent coverage probabilities are 0.0% , 90.3% , 56.7% and 45.5% (Table 6). Using copula model 2, where covariates were included for the hazard rates and the association parameter, the coverage probability is around the nominal level for each copula, 94.6% , 95.1% , 94.5% and 95.2% (Table 6). We observe a reduction in the bias and MSE of the non-terminal event’s hazard ratio of the age group in copula model 2, as compared to the Cox model. This explains the discrepancy in the findings between the Cox model and the copula model in the real data analysis.

For the hazard ratios of sex and donor type for the non-terminal event, both the Cox model and copula model 2 result in coverage probabilities close to the nominal level. However, the results from the copula models show slight reductions in bias and MSE. For example, when using the Cox model for data generated from the Normal copula, the bias and MSE of the hazard ratio are 0.240 and 0.062 , respectively, for the non-terminal event with covariate age group. Comparing this to copula model 2, we find the bias to be −0.003 and the MSE to be 0.007 .

**Table 6.** Results of simulation study.

	Normal			Clayton			Frank			Gumbel		
Estimand	Bias	CP	MSE	Bias	CP	MSE	Bias	CP	MSE	Bias	CP	MSE
**Cox model**												
${\text{HR}}_{NT},\text{age}$	0.240	11.7	0.062	0.552	0.0	0.309	0.505	0.0	0.260	0.223	18.9	0.055
${\text{HR}}_{NT},\text{sex}$	−0.016	94.3	0.006	−0.012	95.2	0.006	−0.026	94.0	0.007	−0.013	94.8	0.006
${\text{HR}}_{NT},\text{donor}$	−0.019	92.8	0.003	−0.019	94.0	0.003	−0.024	93.0	0.003	−0.006	94.4	0.003
${\text{HR}}_{T},\text{age}$	−0.010	95.8	0.079	0.000	94.6	0.086	−0.012	95.4	0.080	−0.004	96.4	0.074
${\text{HR}}_{T},\text{sex}$	−0.001	95.1	0.005	−0.005	94.7	0.005	−0.005	94.8	0.005	0.000	95.5	0.005
${\text{HR}}_{T},\text{donor}$	0.002	95.2	0.002	0.000	94.9	0.002	−0.001	95.5	0.002	0.001	94.8	0.002
**Copula model 1**												
${\text{HR}}_{NT},\text{age}$	−0.155	0.0	0.024	−0.042	90.3	0.012	−0.147	56.7	0.030	−0.146	45.5	0.027
${\text{HR}}_{NT},\text{sex}$	0.075	100.0	0.006	0.004	95.0	0.005	0.007	94.9	0.005	0.004	94.6	0.005
${\text{HR}}_{NT},\text{donor}$	−0.018	100.0	0.000	−0.007	92.5	0.003	−0.002	94.0	0.002	0.000	94.2	0.003
${\text{HR}}_{T},\text{age}$	−0.042	100.0	0.002	0.397	76.9	0.252	0.144	94.6	0.105	−0.132	91.2	0.083
${\text{HR}}_{T},\text{sex}$	0.046	100.0	0.002	0.017	95.4	0.004	−0.001	95.3	0.004	−0.002	94.9	0.004
${\text{HR}}_{T},\text{donor}$	−0.051	100.0	0.003	0.028	92.4	0.003	0.003	95.6	0.002	−0.003	94.6	0.002
**Copula model 2**												
${\text{HR}}_{NT},\text{age}$	−0.003	94.6	0.007	−0.002	95.1	0.009	−0.002	94.5	0.010	−0.003	95.2	0.007
${\text{HR}}_{NT},\text{sex}$	−0.003	94.6	0.006	−0.001	95.9	0.005	−0.002	95.3	0.005	−0.003	95.4	0.006
${\text{HR}}_{NT},\text{donor}$	0.002	94.1	0.003	0.002	93.6	0.002	0.002	94.0	0.003	0.002	94.4	0.003
${\text{HR}}_{T},\text{age}$	−0.009	95.7	0.077	−0.011	95.1	0.080	−0.014	95.0	0.093	−0.004	95.9	0.073
${\text{HR}}_{T},\text{sex}$	−0.001	95.1	0.004	−0.002	95.5	0.004	−0.001	94.6	0.005	0.000	94.8	0.004
${\text{HR}}_{T},\text{donor}$	0.002	95.1	0.002	0.001	94.0	0.002	0.002	94.6	0.002	0.002	94.7	0.002
$\beta_{0}$	0.005	94.3	0.005	0.017	95.5	0.031	0.018	94.2	0.253	0.066	96.6	0.123
$\beta_{1}$	−0.006	96.0	0.006	−0.018	96.2	0.032	−0.056	95.6	0.557	−0.055	96.9	0.129
$\beta_{2}$	−0.004	95.4	0.007	−0.004	95.1	0.025	−0.033	94.9	0.445	−0.007	96.4	0.077
$\beta_{3}$	0.002	96.0	0.009	−0.005	94.6	0.033	−0.030	95.0	0.839	0.014	95.6	0.094

Copula model 1 represents the copula model with the marginal hazard rates depending on the covariates. Copula model 2 represents the copula model with both the marginal hazard rates and association parameters depending on the covariates. Data are generated from the Normal, Clayton, Frank and Gumbel copulas and the true copula distribution is used for estimation. We generate 1000 data sets with 3000 individuals in each. The non-terminal hazard ratio for each covariate is given by ${\text{HR}}_{NT,\text{covariate}}$ , and the terminal hazard ratio for each covariate is given by ${\text{HR}}_{T,\text{covariate}}$ . MSE refers to the mean squared error and CP refers to the coverage probability, given as a percentage.

The results for the hazard ratios of the terminal event are similar between the Cox model, copula models 1 and 2 with coverage probability close to 95% . This is expected, because the terminal event cannot be censored by the non-terminal event. However, using copula model 1 the coverage probability is 90.3 %, for the hazard ratio of age group for the non-terminal event, where data are generated from the Clayton copula. When including the covariate age group in the association parameter, in copula model 2, we find the coverage probability increases to 95.1 %.

### 5.5. Results of simulation study 2: Effects of the misspecification of survival distributions

We evaluate the use of alternative survival distributions and the effects of misspecification of the survival distribution. For the simulation studies investigating misidentification, we simulate data mimicking the real data set using four different copula distributions, with true values given in Supplemental Material Table S2. We also evaluate the use of AIC to select the survival distributions. We simulate data with the following combinations of survival distributions and copula functions, Exponential, Weibull and Gompertz survival distributions and Normal, Clayton, Gumbel and Frank copulas.

The results of simulation study 2 are given in Supplemental Material Table S6. Here we give a summary of findings from this simulation study. Our simulation study shows that AIC can be used to select the survival distributions. The survival distribution is chosen correctly in almost 100% cases for the Weibull distribution, roughly 91% or above for Gompertz distribution, and around 78% or above for Exponential distribution. Where the underlying Exponential distribution is not correctly identified, about 10% of the times the survival distribution is misspecified as Weibull and about 10% as Gompertz distribution. However, the estimation is in general robust to the misidentification of the Exponential distributions by Weibull or Gompertz distribution. The summary of performance of the models selected by the lowest AIC shows that the coverage probability is close to the nominal level.

## 6. Discussion

We propose a set of copula regression models to allow both the hazard rates and the association between times to non-terminal and terminal events to vary by covariates. We use conditional copulas^9^ with predictors for the hazard rates of the semi-competing risk events and the association parameters. The advantage of our proposed method is the estimation of the hazard ratios by taking into account the correlation between the semi-competing risks and the flexibility of using a variety of copulas to describe different patterns in the relationship between the survival endpoints.

Our work demonstrates the importance of considering the correlation between the semi-competing risks. In the real data analysis, different conclusions were found for the effect of age group, where Cox model showed older age was associated with lower risk of graft failure (HR: 0.911, 95% CI: 0.872–0.952), while our proposed copula models showed older age was associated with higher risk of graft failure from all copula models. The estimated hazard ratio from Frank copula Weibull survival model, which has the lowest AIC, is 1.202 with 95% CI: 1.152–1.251 (Table 5). We conduct simulation studies to assess the performance of the copula regression models and to evaluate the use of AIC to choose survival distributions. The estimation of the hazard ratio for the non-terminal event is improved by using copula model 2, with coverage probability around the nominal level, compared to using the Cox model which has coverage probability 0% in some scenarios (Table 6). These results corroborate the findings from Leffondré et al.^23^ For the non-terminal hazard ratio, we found a reduction in bias and MSE using the copula regression models compared to the Cox model. Our work highlights the importance of acknowledging the semi-competing risk when analysing the effect of a covariate on an endpoint, where the covariate has a strong effect on the competing risk.

We have considered the effect of misspecification of survival distributions. Our simulation studies show that the AIC can be used to select the survival distributions in the majority of cases. In the remaining cases, misspecification is more likely to occur when the underlying distribution is Exponential or Gompertz. However, in our simulation studies, miss-specifying these two distributions by a Weibull distribution still holds good properties in terms of the parameter estimates. Further research may also investigate other parametric survival distributions or the use of non-parametric methods to model the marginal survival functions.

We have used a full maximum likelihood approach for estimating the model parameters. We acknowledge the two-stage estimation procedures for the association parameter may be more efficient for copula survival models.^24^ In the two-stage approach, the association parameter and the parameters in the marginal survival distributions are estimated separately. In stage 1, parameters in the marginal distributions are estimated assuming they are independent. In stage 2, the association parameter is estimated by fixing the two marginal distributions at the estimates from stage 1. This approach ignores the dependency structure in stage 1, however, it offers the advantage of being practically efficient, especially when the models become more complex. The application of the two-stage approach in our proposed models and the comparison with the one-stage full maximum likelihood approach is a topic for future research. Another possible extension could be to estimate the marginal survival function using non-parametric methods^25^ or Cox proportional hazards model.^26^ It would be also of interest to investigate the use of grid search methods to find starting values for optimising the likelihood function.

In this article, we have extended our recent work in bivariate semi-competing risk models by including covariates. Another topic of future research could be to extend the model to include frailty terms to account for unmeasured covariates. Since we have bivariate semi-competing risk data, this extension will require to model the frailty terms by using a bivariate distribution to account for the potential correlation between the two frailty terms.

We have used Exponential, Weibull and Gompertz distributions to illustrate the methods and applications of our proposed models. However, this can be readily extended to other parametric survival distributions. We have assessed the performance of our proposed copula survival models using simulation studies and compare between them using AIC. Assessing the goodness-of-fit of the copula survival models may be developed in future research.

As this article focuses on describing copula regression models with binary covariates, the dichotomous age groups ( >50 years or ≤50 years) representing younger and older age were included for illustration purpose. We acknowledge that age should be best analysed with more categories or as a continuous variable. In our follow-up work, we are developing copula regression models to include more categories for age or include age as a continuous variable.

Further research may investigate the inclusion of continuous and categorical covariates. We have considered a linear function to incorporate covariates into the hazard rates and association parameter, however, alternatives may be considered. Acar et al.^27^ have developed methods to compare potential functions that describe the association parameter of the copula function’s relationship with the covariate by using a generalised likelihood ratio test. We have used available case analysis to illustrate the application of our methods. Future research can use multiple imputation to deal with missing data when using our proposed methods. This could be time consuming when the normal copula model is used.

As can be seen in Tables 3 to 5, it takes 2 to 4 h to optimise the likelihood for normal copula models. Future research may investigate how to speed up the optimisation process for the analysis of a single data set, and the use of parallel computing in R for conducting simulation studies.

## Supplemental Material

sj-pdf-1-smm-10.1177_09622802231188516 - Supplemental material for Bivariate copula regression models for semi-competing risks Supplemental material, sj-pdf-1-smm-10.1177_09622802231188516 for Bivariate copula regression models for semi-competing risks by Yinghui Wei, Małgorzata Wojtyś, Lexy Sorrell and Peter Rowe in Statistical Methods in Medical Research
