# Supplementary material for: Bivariate copula regression models for semi-competing risks
Source: Stat Methods Med Res. 2023 Aug 9;32(10):1902–18. doi: 10.1177/09622802231188516 (PMC10563377; doi:10.1177/09622802231188516)
Supplement: sj-pdf-1-smm-10.1177_09622802231188516 - Supplemental material for Bivariate copula regression models for semi-competing risks [file sj-pdf-1-smm-10.1177_09622802231188516.pdf]

Supplementary Material for **"Bivariate copula regression models for semi-competing risks"**

by Yinghui Wei, Małgorzata Wojtyś, Lexy Sorrell and Peter Rowe

1 The Delta method

Based on the Delta method (van der Vaart (2000), p. 25), for a univariate function of a random variable X, $f(X)$, the variance can be approximated by

$$\text{Var}[f(X)] = (f'(\mu_x))^2 \, \text{Var}(X), \tag{1}$$

where $\mu_x = E(X)$.
For a bivariate function of random variables X and Y, $f(X,Y)$, the variance can be approximated by

$$\text{Var}[f(X,Y)] = (f'_x(\boldsymbol{\mu}))^2 \, \text{Var}(X) + 2f'_x(\boldsymbol{\mu})f'_y(\boldsymbol{\mu})\text{Cov}(X,Y) + \left(f'_y(\boldsymbol{\mu})\right)^2 \text{Var}(Y), \tag{2}$$

where $\boldsymbol{\mu} = (\mu_x, \mu_y) = (E(X), E(Y))$.
For a p-variate function $f(X_1, \ldots, X_p)$, the variance can be approximated by

$$\text{Var}[f(X_1, \ldots, X_p)] = \nabla f(\boldsymbol{\mu})^T \boldsymbol{\Sigma}_{\boldsymbol{X}} \nabla f(\boldsymbol{\mu}), \tag{3}$$

where $\boldsymbol{\Sigma}_{\boldsymbol{X}}$ is the covariance matrix of the random vector $\boldsymbol{X} = (X_1, \ldots, X_p)$ and $\nabla f(\boldsymbol{\mu})$ is the gradient of f with $\boldsymbol{\mu} = (E(X_1), \ldots, E(X_p))$.

1.1 Variance of the hazard ratio

To calculate the variance of hazard ratios, we use the Delta method. For the Exponential, Weibull and Gompertz distributions, the hazard ratio is given in the same exponential form. Thus, we find the approximation of the variance of $f(X) = \exp(X)$, which is given by

$$\text{Var}[f(X)] = \exp(2\mu)\text{Var}(X).$$

1.2 Variance of the Normal association parameter

1.2.1 One covariate

When the Normal copula parameter ρ varies with covariate, W_1, we assume $\rho = \dfrac{\exp(2(b_0 + b_1 W_1)) - 1}{\exp(2(b_0 + b_1 W_1)) + 1}$ therefore we want an approximation for the variance of the function $f(b_0, b_1) = \dfrac{\exp(2(b_0 + b_1 W_1)) - 1}{\exp(2(b_0 + b_1 W_1)) + 1}$. The derivatives are given by the following,

$$f'_{\beta_0} = \frac{\partial}{\partial b_0}\left(\frac{\exp(2(b_0 + b_1 W_1)) - 1}{\exp(2(b_0 + b_1 W_1)) + 1}\right) = \frac{4\exp(2(b_0 + b_1 W_1))}{(\exp(2(b_0 + b_1 W_1)) + 1)^2}, \tag{4}$$

$$f'_{b_1} = \frac{\partial}{\partial b_1}\left(\frac{\exp(2(b_0 + b_1 W_1)) - 1}{\exp(2(b_0 + b_1 W_1)) + 1}\right) = \frac{4W_1\exp(2(b_0 + b_1 W_1))}{(\exp(2(b_0 + b_1 W_1)) + 1)^2}. \tag{5}$$

Substituting (4) and (5) into equation (2), the variance of $\hat{\rho}$ is approximated by,

$$\mathrm{Var}(\hat{\rho}) = \frac{16\exp(4(b_0 + b_1 W_1))}{(\exp(2(b_0 + b_1 W_1)) + 1)^4}\left[\mathrm{Var}(\hat{b}_0) + 2W_1\mathrm{Cov}(\hat{b}_0, \hat{b}_1) + W_1^2\mathrm{Var}(\hat{b}_1)\right]. \tag{6}$$

1.2.2 Multiple covariates

In general, when p covariates $W_1, \ldots, W_p$ are considered, we assume $\rho = \dfrac{\exp(2(b_0 + b_1 W_1 + \ldots + b_p W_p)) - 1}{\exp(2(b_0 + b_1 W_1 \ldots + b_p W_p)) + 1}$. Using vector notation, we define the function

$$f(\boldsymbol{b}) = \frac{\exp\left(2\boldsymbol{b}^T\boldsymbol{W}\right) - 1}{\exp\left(2\boldsymbol{b}^T\boldsymbol{W}\right) + 1},$$

where $\boldsymbol{b} = (b_0, b_1, \ldots, b_p)^T$ and $\boldsymbol{W} = (1, W_1, \ldots, W_p)^T$.
The partial derivative with respect to b_k is given by

$$f'_{b_k} = \frac{\partial}{\partial b_k} f(\boldsymbol{b}) = \frac{4W_k \exp(2\boldsymbol{b}^T\boldsymbol{W})}{(\exp(2\boldsymbol{b}^T\boldsymbol{W}) + 1)^2} \tag{7}$$

for $k = 0, 1, \ldots, p$. Therefore, using equation (2), the variance of $\hat{\rho}$ is approximated by

$$\mathrm{Var}(\hat{\rho}) = \nabla f(\boldsymbol{b})^T \boldsymbol{\Sigma}_{\hat{\boldsymbol{b}}} \nabla f(\boldsymbol{b}),$$

where $\boldsymbol{\Sigma}_{\hat{\boldsymbol{b}}}$ is the covariance matrix of $\hat{\boldsymbol{b}}$ and $\nabla f(\boldsymbol{b}) = \left(f'_{b_0}, f'_{b_1}, \ldots, f'_{b_p}\right)^T$ as given in (7).

1.3 Variance of the Clayton association parameter

1.3.1 One covariate

For the Clayton copula, we assume $\theta = \exp(b_0 + b_1 W_1)$, therefore we want an approximation for the variance of the function $f(b_0, b_1) = \exp(b_0 + b_1 W_1)$. The derivatives are given by,

$$f'_{b_0} = \frac{\partial \exp(b_0 + b_1 W_1)}{\partial b_0} = \exp(b_0 + b_1 W_1), \tag{8}$$

$$f'_{b_1} = \frac{\partial \exp(b_0 + b_1 W_1)}{\partial b_1} = W_1 \exp(b_0 + b_1 W_1). \tag{9}$$

Substituting (8) and (9) into equation (2), the variance is approximated by,

$$\mathrm{Var}(\hat{\theta}) = \exp(2(b_0 + b_1 W_1))[\mathrm{Var}(\hat{b}_0) + 2W_1\mathrm{Cov}(\hat{b}_0, \hat{b}_1) + W_1^2\mathrm{Var}(\hat{b}_1)]. \tag{10}$$

1.3.2 Multiple covariates

In general, when p covariates $W_1, \ldots, W_p$ are considered, we assume $\theta = \exp(b_0 + b_1 W_1 + \ldots + b_p W_p)$. Using vector notation, we define the function

$$f(\boldsymbol{b}) = \exp\left(\boldsymbol{b}^T \boldsymbol{W}\right),$$

where $\boldsymbol{b} = (b_0, b_1, \ldots, b_p)^T$ and $\boldsymbol{W} = (1, W_1, \ldots, W_p)^T$.
The partial derivative with respect to b_k is given by

$$f'_{b_k} = \frac{\partial}{\partial b_k} f(\boldsymbol{b}) = W_k \exp(\boldsymbol{b}^T \boldsymbol{W}) \tag{11}$$

for $k = 0, 1, \ldots, p$. Therefore, using equation (2), the variance of $\hat{\theta}$ is approximated by

$$\text{Var}(\hat{\theta}) = \nabla f(\boldsymbol{b})^T \boldsymbol{\Sigma}_{\hat{\boldsymbol{b}}} \nabla f(\boldsymbol{b}),$$

where $\boldsymbol{\Sigma}_{\hat{\boldsymbol{b}}}$ is the covariance matrix of $\hat{\boldsymbol{b}}$ and $\nabla f(\boldsymbol{b}) = \left(f'_{b_0}, f'_{b_1}, \ldots, f'_{b_p}\right)^T$ as given in (11).

1.4 Variance of the Gumbel association parameter

1.4.1 One covariate

For the Gumbel copula, we assume $\theta = \exp(b_0 + b_1 W_1) + 1$, therefore we want an approximation for the variance of the function $f(b_0, b_1) = \exp(b_0 + b_1 W_1) + 1$. The derivatives are given by,

$$f'_{b_0} = \frac{\partial \exp(b_0 + b_1 W_1) + 1}{\partial b_0} = \exp(b_0 + b_1 W_1), \tag{12}$$

$$f'_{b_1} = \frac{\partial \exp(b_0 + b_1 W_1) + 1}{\partial b_1} = W_1 \exp(b_0 + b_1 W_1). \tag{13}$$

Substituting (12) and (13) into equation (2), the variance is approximated by,

$$\text{Var}(\hat{\theta}) = \exp(2(b_0 + b_1 W_1))[\text{Var}(\hat{b}_0) + 2W_1 \text{Cov}(\hat{b}_0, \hat{b}_1) + W_1^2 \text{Var}(\hat{b}_1)]. \tag{14}$$

1.4.2 Multiple covariates

In general, when p covariates $W_1, \ldots, W_p$ are considered, we assume $\theta = \exp(b_0 + b_1 W_1 + \ldots + b_p W_p) + 1$. Using vector notation, we define the function

$$f(\boldsymbol{b}) = \exp\left(\boldsymbol{b}^T \boldsymbol{W}\right) + 1,$$

where $\boldsymbol{b} = (b_0, b_1, \ldots, b_p)^T$ and $\boldsymbol{W} = (1, W_1, \ldots, W_p)^T$.
The partial derivative with respect to b_k is given by

$$f'_{b_k} = \frac{\partial}{\partial b_k} f(\boldsymbol{b}) = W_k \exp(\boldsymbol{b}^T \boldsymbol{W}) \tag{15}$$

for $k = 0, 1, \ldots, p$. Therefore, using equation (2), the variance of $\hat{\theta}$ is approximated by

$$\mathrm{Var}(\hat{\theta}) = \nabla f(\boldsymbol{b})^T \boldsymbol{\Sigma}_{\hat{\boldsymbol{b}}} \nabla f(\boldsymbol{b}),$$

where $\boldsymbol{\Sigma}_{\hat{\boldsymbol{b}}}$ is the covariance matrix of $\hat{\boldsymbol{b}}$ and $\nabla f(\boldsymbol{b}) = \left(f'_{b_0}, f'_{b_1}, \ldots, f'_{b_p}\right)^T$ as given in (15).

2 Regression coefficients estimated from copula regression models

Parameter	Normal-Exponential	Clayton-Exponential	Frank-Exponential	Gumbel-Exponential
Graft failure				
a_0	−3.30 (−3.33, −3.27)	−3.28 (−3.31, −3.24)	−3.28 (−3.31, −3.24)	−3.33 (−3.36, −3.29)
a_1	0.11 (0.07, 0.16)	0.32 (0.29, 0.36)	0.31 (0.27, 0.35)	0.13 (0.08, 0.17)
a_2	0.01 (−0.03, 0.06)	0.01 (−0.03, 0.05)	0.00 (−0.04, 0.04)	0.01 (−0.03, 0.06)
a_3	−0.51 (−0.56, −0.46)	−0.53 (−0.58, −0.48)	−0.53 (−0.58, −0.48)	−0.51 (−0.56, −0.46)
Death				
c_0	−4.15 (−4.19, −4.11)	−4.09 (−4.13, −4.05)	−4.08 (−4.12, −4.04)	−4.16 (−4.20, −4.11)
c_1	1.32 (1.27, 1.37)	1.35 (1.31, 1.40)	1.35 (1.31, 1.40)	1.30 (1.25, 1.35)
c_2	−0.11 (−0.15, −0.06)	−0.07 (−0.11, −0.03)	−0.07 (−0.11, −0.03)	−0.11 (−0.15, −0.06)
c_3	−0.65 (−0.71, −0.59)	−0.62 (−0.67, −0.56)	−0.63 (−0.68, −0.57)	−0.64 (−0.70, −0.58)
Association				
b_0	0.35 (0.32, 0.38)	0.39 (0.29, 0.50)	3.04 (2.75, 3.33)	−2.30 (−2.44, −2.15)
b_1	0.28 (0.25, 0.32)	1.09 (0.98, 1.20)	5.07 (4.60, 5.54)	1.35 (1.20, 1.51)
b_2	0.02 (−0.01, 0.06)	0.14 (0.04, 0.24)	0.35 (−0.06, 0.76)	0.06 (−0.08, 0.19)
b_3	0.03 (−0.03, 0.08)	0.53 (0.38, 0.67)	0.86 (0.21, 1.51)	−0.04 (−0.23, 0.15)

Table S1: Regression coefficients estimated from copula Exponential survival models. Here $a_i (i = 0, 1, 2, 3)$ are regression coefficients for the hazard function for graft failure, $c_i (i = 0, 1, 2, 3)$ are regression coefficients for the hazard function for death, and $b_i (i = 0, 1, 2, 3)$ are regression coefficients for the association parameter between graft failure and death.

Parameter	Normal-Weibull	Clayton-Weibull	Frank-Weibull	Gumbel-Weibull
Graft failure				
a_0	-2.51 (-2.56, -2.47)	-2.58 (-2.62, -2.54)	-2.56 (-2.60, -2.52)	-2.56 (-2.60, -2.51)
a_1	0.01 (-0.04, 0.05)	0.19 (0.15, 0.23)	0.18 (0.14, 0.22)	-0.01 (-0.05, 0.03)
a_2	0.02 (-0.02, 0.06)	0.02 (-0.02, 0.07)	0.01 (-0.03, 0.05)	0.02 (-0.02, 0.06)
a_3	-0.56 (-0.61, -0.51)	-0.58 (-0.63, -0.53)	-0.58 (-0.63, -0.53)	-0.54 (-0.59, -0.49)
Death				
c_0	-4.14 (-4.20, -4.08)	-4.03 (-4.09, -3.96)	-4.03 (-4.09, -3.97)	-4.00 (-4.06, -3.94)
c_1	1.31 (1.26, 1.35)	1.31 (1.26, 1.35)	1.32 (1.27, 1.36)	1.28 (1.24, 1.33)
c_2	-0.10 (-0.14, -0.06)	-0.08 (-0.12, -0.04)	-0.08 (-0.12, -0.04)	-0.10 (-0.14, -0.06)
c_3	-0.65 (-0.71, -0.59)	-0.64 (-0.70, -0.59)	-0.65 (-0.71, -0.60)	-0.65 (-0.71, -0.59)
Association				
b_0	0.42 (0.38, 0.46)	0.41 (0.29, 0.53)	3.20 (2.89, 3.52)	-1.81 (-1.94, -1.67)
b_1	0.28 (0.24, 0.33)	0.91 (0.78, 1.03)	3.95 (3.49, 4.42)	1.07 (0.94, 1.21)
b_2	0.03 (-0.01, 0.07)	0.16 (0.05, 0.27)	0.17 (-0.25, 0.59)	0.04 (-0.08, 0.17)
b_3	0.03 (-0.03, 0.09)	0.48 (0.31, 0.64)	0.49 (-0.17, 1.16)	-0.04 (-0.22, 0.13)
Shape parameter				
α_1	0.67 (0.65, 0.68)	0.70 (0.69, 0.71)	0.70 (0.68, 0.71)	0.67 (0.66, 0.68)
α_2	1.02 (1.00, 1.04)	0.98 (0.96, 0.99)	0.99 (0.97, 1.01)	0.96 (0.94, 0.98)

Table S2: Regression coefficients estimated from copula Weibull survival models. Here $a_i (i = 0, 1, 2, 3)$ are regression coefficients for the scale parameter in Weibull distribution for graft failure, $b_i (i = 0, 1, 2, 3)$ are regression coefficients for the association parameter between graft failure and death, $c_i (i = 0, 1, 2, 3)$ are regression coefficients for the scale parameter in Weibull distribution for death and $\alpha_j (j = 1, 2)$ are the shape parameters in Weibull distribution.

Parameter	Normal-Gompertz	Clayton-Gompertz	Frank-Gompertz	Gumbel-Gompertz
Graft failure				
a_0	0.02 (−0.02, 0.06)	0.00 (−0.04, 0.04)	0.00 (−0.04, 0.04)	0.01 (−0.03, 0.05)
a_1	−0.52 (−0.57, −0.47)	−0.52 (−0.57, −0.47)	−0.61 (−0.66, −0.56)	−0.52 (−0.57, −0.47)
a_2	0.34 (0.31, 0.37)	0.41 (0.31, 0.51)	3.92 (3.62, 4.22)	−2.31 (−2.45, −2.17)
a_3	0.28 (0.25, 0.32)	0.95 (0.84, 1.06)	2.07 (1.66, 2.49)	1.40 (1.25, 1.55)
Death				
c_0	0.02 (−0.01, 0.06)	0.16 (0.06, 0.26)	0.64 (0.23, 1.04)	−0.02 (−0.15, 0.12)
c_1	0.02 (−0.03, 0.07)	0.29 (0.12, 0.46)	0.42 (−0.24, 1.09)	−0.10 (−0.29, 0.10)
c_2	−4.56 (−4.61, −4.50)	−4.33 (−4.38, −4.28)	−4.33 (−4.38, −4.28)	−4.57 (−4.63, −4.52)
c_3	1.43 (1.38, 1.47)	1.37 (1.32, 1.42)	1.37 (1.33, 1.42)	1.41 (1.37, 1.46)
Association				
b_0	−0.11 (−0.16, −0.07)	−0.10 (−0.14, −0.06)	−0.08 (−0.12, −0.04)	−0.12 (−0.16, −0.07)
b_1	−0.59 (−0.65, −0.53)	−0.59 (−0.64, −0.53)	−0.70 (−0.76, −0.64)	−0.58 (−0.64, −0.52)
b_2	−0.02 (−0.02, −0.01)	0.00 (0.00, 0.00)	−0.01 (−0.01, −0.01)	−0.02 (−0.03, −0.02)
b_3	0.05 (0.05, 0.06)	0.03 (0.03, 0.04)	0.04 (0.04, 0.04)	0.06 (0.05, 0.06)
Shape parameter				
γ_1	−3.20 (−3.24, −3.16)	−3.26 (−3.30, −3.21)	−3.19 (−3.23, −3.14)	−3.20 (−3.25, −3.16)
γ_2	0.08 (0.03, 0.12)	0.26 (0.22, 0.30)	0.21 (0.17, 0.25)	0.06 (0.02, 0.11)

Table S3: Regression coefficients estimated from copula Gompertz survival models. Here $a_i (i = 0, 1, 2, 3)$ are regression coefficients for the hazard function for graft failure, $c_i (i = 0, 1, 2, 3)$ are regression coefficients for the hazard function for death, $b_i (i = 0, 1, 2, 3)$ are regression coefficients for the association parameter between graft failure and death, and $\gamma_j (j = 1, 2)$ are the shape parameters in Gompertz distribution.

3 True values used in simulation studies

	Copula			
Parameter	Normal	Clayton	Frank	Gumbel
Graft failure				
a_0	-3.30	-3.28	-3.27	-3.33
a_1	0.11	0.32	0.31	0.13
a_2	0.02	0.00	0.00	0.00
a_3	-0.51	-0.53	-0.53	-0.51
Death				
c_0	-4.15	-4.09	-4.08	-4.16
c_1	1.32	1.35	1.35	1.30
c_2	-0.11	-0.07	-0.07	-0.11
c_3	-0.65	-0.62	-0.62	-0.64
Association				
b_0	0.35	0.39	3.06	-2.30
b_1	0.28	1.09	5.07	1.35
b_2	0.00	0.14	0.00	0.00
b_3	0.00	0.53	0.86	0.00

Table S4: True values used in simulation study 1: comparison of the Cox model and Copula models.

	Copula			
Parameter	Normal	Clayton	Frank	Gumbel
Exponential survival distribution				
a_0	−3.440	−3.420	−3.420	−3.460
a_1	0.170	0.380	0.370	0.180
c_0	−4.360	−4.28	−4.270	−4.360
c_1	1.390	1.410	1.410	1.360
b_0	0.370	0.620	3.440	−2.240
b_1	0.290	1.040	5.230	1.350
Weibull survival distribution				
α_{nt}	0.670	0.710	0.700	0.680
α_t	1.030	0.980	0.990	0.970
$\beta_{nt,0}$	−2.680	−2.750	−2.740	−2.720
$\beta_{nt,1}$	0.070	0.260	0.260	0.050
$\beta_{t,0}$	−4.380	−4.300	−4.250	−4.230
$\beta_{t,1}$	1.370	1.330	1.390	1.350
b_0	0.450	0.550	3.540	−1.770
b_1	0.280	0.740	4.140	1.060
Gompertz survival distribution				
γ_{nt}	0.001	0.004	0.001	0.001
γ_t	0.060	0.040	0.040	0.060
$\lambda_{nt,0}$	−3.370	−3.450	−3.420	−3.370
$\lambda_{nt,1}$	0.140	0.360	0.350	0.110
$\lambda_{t,0}$	−4.790	−4.550	−4.620	−4.820
$\lambda_{t,1}$	1.490	1.460	1.510	1.490
b_0	0.360	0.580	3.280	−2.250
b_1	0.280	0.900	4.050	1.310

Table S5: True values used in simulation study 2: misspecification of the survival distribution.

4 Results of Simulation Study 2

Underlying distribution	HR_{NT}			HR_T			Reference ρ			Covariate ρ			Percentage Chosen		
	Bias	CP	MSE	Bias	CP	MSE	Bias	CP	MSE	Bias	CP	MSE	Exp	Weibull	Gompertz
**Normal**															
Exponential	0.001	95.3	0.000	−0.018	94.1	0.1	−0.001	95.4	0.000	0.002	95.0	0.000	79.8	10.4	9.8
Weibull	0.007	93.0	0.000	0.012	97.0	0.1	0.003	97.0	0.000	0.003	97.0	0.000	0.0	100.0	0.0
Gompertz	0.066	92.0	0.300	0.252	94.0	0.6	0.033	96.0	0.200	0.029	90.0	0.200	0.0	2.0	98.0
**Clayton**															
Exponential	0.001	93.8	0.01	0.014	93.8	0.097	−0.001	95.8	0.002	0	94	0	78.8	11	10.2
Weibull	−0.002	94.6	0.008	0.009	93.9	0.087	−0.002	95.8	0.002	−0.001	94.7	0	0	100	0
Gompertz	0.001	93.3	0.011	0.013	94.5	0.097	−0.001	94.6	0.002	0	94.2	0	0	3.1	96.9
**Frank**															
Exponential	0.002	94.3	0.010	0.011	94.4	0.094	0.000	94.5	0.002	−0.001	94.1	0.000	82.4	14.5	3.1
Weibull	−0.002	94.1	0.008	0.009	94.6	0.09	0.000	94.9	0.002	−0.001	95.1	0.000	0.0	100.0	0.0
Gompertz	−0.014	92.4	0.010	0.031	93.9	0.123	0.033	92.1	0.003	−0.015	89.2	0.001	0.0	8.7	91.3
**Gumbel**															
Exponential	0.168	84.8	0.27	0.018	80.2	0.091	0.001	95.0	0.001	−0.003	95.0	0.002	79.5	9.6	10.9
Weibull	−0.002	95.1	0.006	0.021	94.5	0.085	0.001	96.0	0.001	−0.002	94.9	0.001	0.1	99.8	0.1
Gompertz	−0.003	95.7	0.006	0.017	94.9	0.109	0.002	96.5	0.001	−0.004	93.8	0.002	0.0	0.2	99.8

Table S6: Simulation study for the misspecification of survival distributions. Results of the simulation study with data generated from either the Exponential, Weibull or Gompertz survival distributions and the Normal, Clayton, Frank and Gumbel copulas. The model with the lowest AIC is chosen and results are recorded for 1000 data sets with 3000 individuals in each. The MSE refers to the mean squared error and CP refers to the coverage probability, given as a percentage.

References

van der Vaart, A. W. (2000). *Asymptotic Statistics*. Cambridge University Press.
